# One Medicine, One Acupuncture

**DOI:** 10.3390/ani2030395

**Published:** 2012-08-29

**Authors:** Narda G. Robinson

**Affiliations:** CSU Center for Comparative and Integrative Pain Medicine, College of Veterinary Medicine and Biomedical Sciences, Colorado State University, 300 West Drake Road, Fort Collins, CO 80523, USA; E-Mail: Narda.Robinson@colostate.edu; Tel.: +1-970-297-4202; Fax: +1-970-297-1275

**Keywords:** acupuncture, veterinary acupuncture, anatomy, neuromodulation, comparative anatomy, translational research, One Medicine, One Acupuncture

## Abstract

**Simple Summary:**

“One Acupuncture”, modeled after “One Medicine”, embodies a system of translational acupuncture built upon science and hypothesis-driven research. Forging a synthesis between human and veterinary acupuncture requires consistency in point location across species so that meaningful comparisons can be made. The human acupuncture network provides a template of well-studied neurovascular sites that have changed little over the years, in comparison to their veterinary counterparts. This paper identifies disparities that remain. Reconciling inconsistencies will bolster the ability for researchers and clinicians to better understand and interpret findings from acupuncture studies on various species so that more can benefit from these insights.

**Abstract:**

“One Acupuncture”, like “One Medicine”, has the potential to improve research quality and clinical outcomes. However, while human acupuncture point locations have remained largely consistent over time, the veterinary versions remain imprecise and variable. Establishing anatomical criteria for veterinary acupuncture atlases in keeping with the human template will create congruence across species, benefiting both research and practice. Anatomic criteria for points based on objectively verifiable structures will facilitate translational research. Functionally comparative innervation, in particular, should be similar between species, as the nerves initiate and mediate physiologic changes that result from point stimulation. If researchers choose points that activate different nerves in one species than in another, unpredictable outcomes may occur. Variability in point placement will impede progress and hamper the ability of researchers and clinicians to make meaningful comparisons across species. This paper reveals incongruities that remain between human and veterinary acupuncture points, illustrating the need to analyze anatomical characteristics of each point to assure accuracy in selecting transpositional acupuncture locations.

## 1. Introduction

“The dissension concerning acupoint location and name within and between the [TCVM] and [transpositional] schools makes communication among veterinary acupuncturists extremely difficult. This is a stumbling block in the advance of veterinary acupuncture’s acceptance as a true clinical science. Veterinarians and non-veterinary animal researchers should never assume any common knowledge regarding acupoint location in the reporting of clinical cases and research involving animal acupuncture. It is not enough to simply state which point location method was followed because there are many disparities within each school. Every acupoint location, name, and indication must be explicitly described in each report—preferably with charts as well as text—regardless of the methods used to locate and name the point. In doing so, we may eventually gather enough empirical and scientific data to ... map universally accepted veterinary acupuncture point charts with a common nomenclature [1].” 

“Between animal and human medicine there is no dividing line—nor should there be. The object is different but the experience obtained constitutes the basis of all medicine [2].” 

“The need for a holistic, collaborative approach – one strategy to better understand and address the contemporary health issues created by the convergence of human, animal, and environmental domains – is the concept of One Health [3].” 

## 2. Opening Communication Channels

The bridges being built between human and veterinary medicine allow the value of translational medicine to benefit all [4,5,6]. The “One Medicine” ideal would eliminate the fragmentation that separates private practitioners from academicians in their “silos of specialists”. It also lessens the gap that artificially divides physicians and veterinarians. Similarly, the nascent “One Acupuncture” paradigm invites contributions from researchers, clinicians, and practicing acupuncturists who share a common language of science and a unified vision to improve healthcare for all, whether their species focus is human or nonhuman. Like One Medicine, One Acupuncture supports efforts to better understand the pros and cons of various interventions across species, promoting advancements through critical assessment of translational techniques. For example, while human acupuncture research on spinal cord injury is somewhat lacking, several studies have examined the value of acupuncture for dogs with similar conditions [7,8,9]. Thus, information derived from one species receiving acupuncture for the treatment of naturally occurring disease may provide insights for applications to other species, facilitating recovery, reducing costs of medical care, and leading to a better quality of life. 

## 3. Promoting Safety with a “One Acupuncture” Framework

A broader perspective is more likely to identify and limit risky techniques. A notable example includes gold bead implants, a method still advocated today [10]. Modeled after Japanese needle embedding, those who practice gold bead implantation permanently embed tens, hundreds, or thousands of metal fragments into tissue and around joints [11,12]. The original technique involved severing acupuncture needles at the skin level to provide “permanent acupuncture”. In the mid-1970s, veterinary acupuncturists in North America decided to modify the technique and place small bits of gold wire deep into tissue through a long spinal needle. They renamed the procedure “gold bead therapy”. Veterinarians now may more commonly implant sterile or nonsterile stainless steel “beads” and gold-plated magnets [13]. 

Papers in human journals warn about permanent needle embedding. One stated, “Despite the fact that such Japanese techniques involving permanent needle retention are neither taught nor practiced in the West, they have contributed significantly to the unfavorable publicity about adverse events [14].” Even as far back as 1976, the Japanese Acupuncture and Moxibustion Association (JAMA) tried to prohibit the practice. For those who did not cease needle implantation, JAMA commented, “Some practitioners do not abide by the recommendation and are ignorant of the results of malpractice.” Authors from a 2007 report in *Chest* stated, “Migration of embedded acupuncture needles is associated with life-threatening consequences. The practice of embedding acupuncture needles is now considered malpractice and is discouraged by professional acupuncture associations [15].” Another author claimed that many more injuries appear in the Japanese literature but the language barrier has prevented this information from becoming more readily known [16]. 

The potential dangers from placing irretrievable metal fragments into patients are many. Implants may migrate to the spinal cord, peritoneal cavity, heart, stomach, liver, breast, brain, bladder, kidney, and colon. Removing implants poses risk and traumatizes tissue. Depending on the metal substrate, implants may activate mast cells, incite inflammation, produce argyria, and lead to contact dermatitis or gold-induced myelotoxicity. Embedded metallic objects may interfere with diagnostic imaging, obscuring diagnoses and wasting resources on artifact-laden images. Had the veterinary acupuncture community learned from the mistakes made by Japanese acupuncturists, innumerable veterinary patients may have been spared the consequences of metal migration and tissue injuries. 

**Figure 1:**
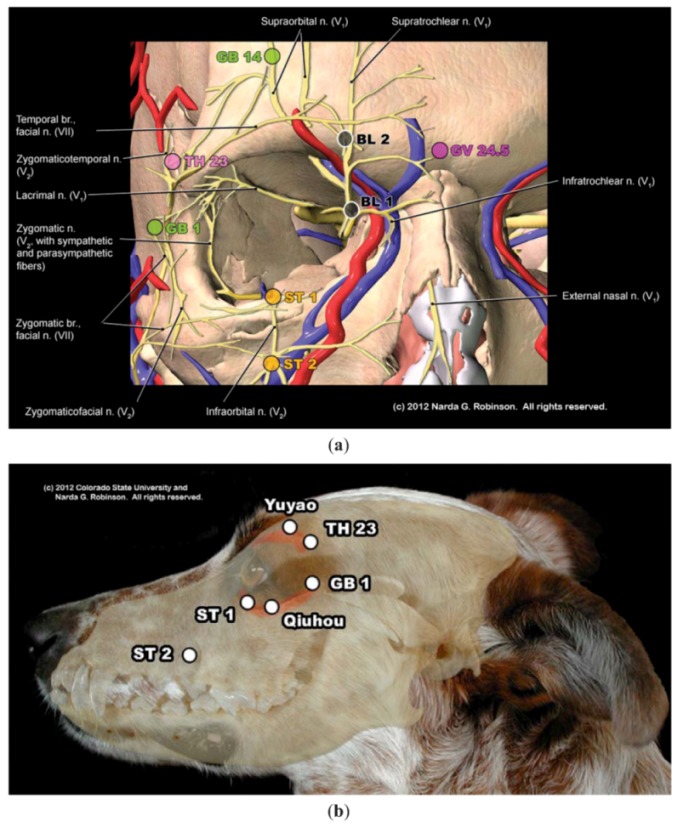
(**a**) This image illustrates the complete bony orbit of the human. Note the relationship of periocular acupuncture points to the orbit, showing how a needle inserted into TH 23 would reach bone and deliver feedback through the needle and to the acupuncturist that a hard surface was reached. In addition, this rendition reveals the complex interplay of trigeminal and facial nerve branches that mediate the ophthalmic benefits of needling TH 23. (Image courtesy of Narda G. Robinson, DO, DVM, MS and Teton NewMedia. From [17]); (**b**) This overlay image of the dog reveals the relationship of the skull to a dog’s external features. Note the hiatus of the orbit between GB 1 and the site just ventral to TH 23. (Image courtesy of Narda G. Robinson, DO, DVM, MS and Colorado State University).

**Figure 2:**
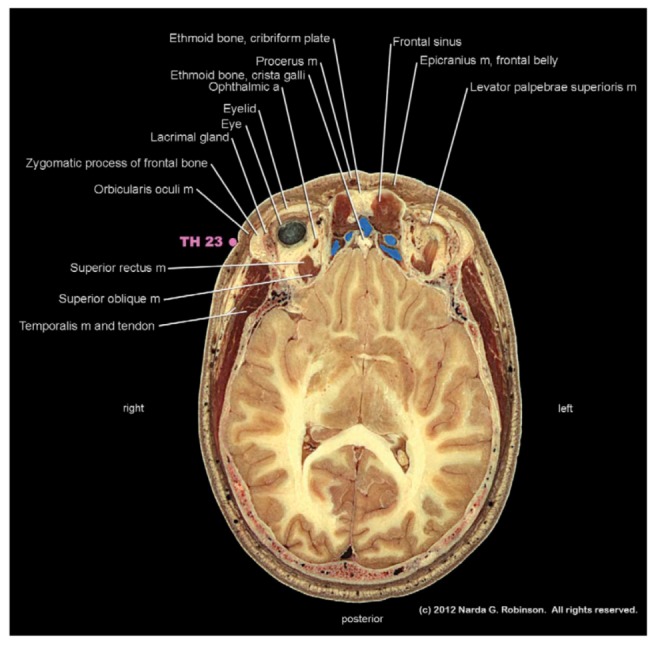
This cross section demonstrates the relationship of acupuncture point TH 23 to the zygomatic process of the frontal bone and the overlying thin layer of orbicularis oculi muscle. The clinical significance of this anatomy pertains to translational safety issues in acupuncture. That is, a human acupuncturist expects to reach bone shortly after penetrating the soft tissues. In contrast, the bony orbit in the dog ends at this site. A needle entering the soft tissue could therefore enter the orbit. (Image courtesy of Narda G. Robinson, DO, DVM, MS and Teton NewMedia. From [17]).

Another way in which a One Acupuncture approach would augment safety pertains to precautions needed when needling over organs or vessels. Practitioners who cross train (*i.e*., gain the ability through appropriate credentialing and lawful licensing to perform both human and veterinary acupuncture) should gain sufficient familiarity with each species’ anatomy in order to avoid injury. One example concerns the treatment of dry eye (keratoconjunctivitis sicca). Both humans and dogs experience this condition; acupuncture studies show value for humans, but there are no studies in dogs. Safely extrapolating point locations from humans to dogs requires knowledge of the differences in periocular anatomy between humans and dogs. That is, most carnivores have an incomplete bony orbital rim while humans and herbivores form complete rings. Compare the orbital anatomy of the human and dog in Figure 1(a,b). Clinically, because only a thin layer of muscle covers the human rim, an acupuncturist expects to encounter hard resistance when the needle meets bone beneath Triple Heater 23 (TH 23, Si Zhu Kong, “Silken Bamboo Hollow”). For example, see the cross section of the human skull at the level of TH 23 in Figure 2). If an acupuncturist expected to reach bone at this site in a dog when needling TH 23, s/he may insert the needle too deeply and penetrate the globe.

## 4. Science as the Foundation for One Acupuncture

Modern medicine’s ability to determine how acupuncture works far outstrips anything the ancient Chinese could have conceived. Through physiologic research and astute observation, myriad somatosomatic, somatovisceral, and somatoautonomic reflexes have come to light since the 1970’s when acupuncture research in the West began in earnest. These reflexes manifest from activation of peripheral nerves in the vicinity of acupuncture points; nerves may transmit sensory, motor, or autonomic signals. Neuromodulation, *i.e*., the normalization of nerve function and promotion of homeostasis and analgesia, explains nearly all of the effects that result from acupuncture [18,19,20,21]. Reliable neuromodulation across species requires similar nerve activation which, in turn, results from consistency in acupuncture point location parameters.

## 5. How Does Needling Influence Nerves?

Beneath the surface, acupuncture needles engage collagen fibers, the connective tissue matrix, nerve endings, and vessels. Deeper needle insertion stimulates mechanoreceptors in muscle tissue. Needle manipulation causes mechanoreceptor and nociceptor activation as well as vasodilation changes and modifications in tissue biochemistry. Over half of all acupuncture points overlie tissue richly supplied with muscle spindles. Examples include Stomach 36 (ST 36, Zu San Li or “Leg Three Miles”) in the cranial (or anterior) tibialis muscle, Large Intestine 4 (LI 4, Hegu or “Union Valley”) in the first dorsal interosseous muscle, and Gallbladder 30 (GB 30, Huan Tiao or “Jumping Round”) over the piriformis muscle [22]. Densely concentrated cutaneous receptors live adjacent to the bases of fingernails on fingers and toes where “ting” or “jing-well” points reside. Another highly sensitive location rich with autonomic fibers is Governor Vessel 26 (GV 26, Shui Gou or “Water Trough”) in the philtrum below the nose.

## 6. Acupuncture Effects and the Central Nervous System

Acupuncture-induced neural signals ascend to the spinal cord. Once there, they influence interneuronal processing of sensory input arriving at the dorsal horn and modulate channel and receptor activities [23,24]. The signal ascends to the brain and activates or deactivates key centers such as the limbic system, cortex, cerebellum, and brainstem [25,26]. Shifts in autonomic tone affect output to the digestive and cardiopulmonary systems. Researchers have traced long-loop pathways from points such as Pericardium 6 (PC 6, Nei Guan or “Inner Pass”), over the median nerve, to the rostral ventrolateral medulla, the arcuate nucleus of the hypothalamus, the ventrolateral periaqueductal gray area of the midbrain, and the medullary raphe [27,28]. In order to compare outcomes of PC 6 stimulation in various species, one should first ensure accurate location of the median nerve and similar relationships with surrounding antebrachial anatomy. The same approach should be followed for every nerve-point dyad of the other 360+ points extrapolated from human to non-human anatomy over the past several decades. Traditional Chinese veterinary medical (TCVM) points, discussed more fully below, would not qualify for a cross-species comparative anatomy investigation because ancient veterinary points shifted considerably in location and number as time progressed while the human system remained relatively consistent [1]. For example, as late as 1604, equine charts listed a mere one dozen points [29], whereas the human set of points exceeds 360. 

## 7. Sources of Confusion in Traditional Chinese Veterinary Medical (TCVM) Point Locations

As noted above, much of the variability in nonhuman points arises from the introduction of TCVM points into the veterinary repertoire. 

**Figure 3:**
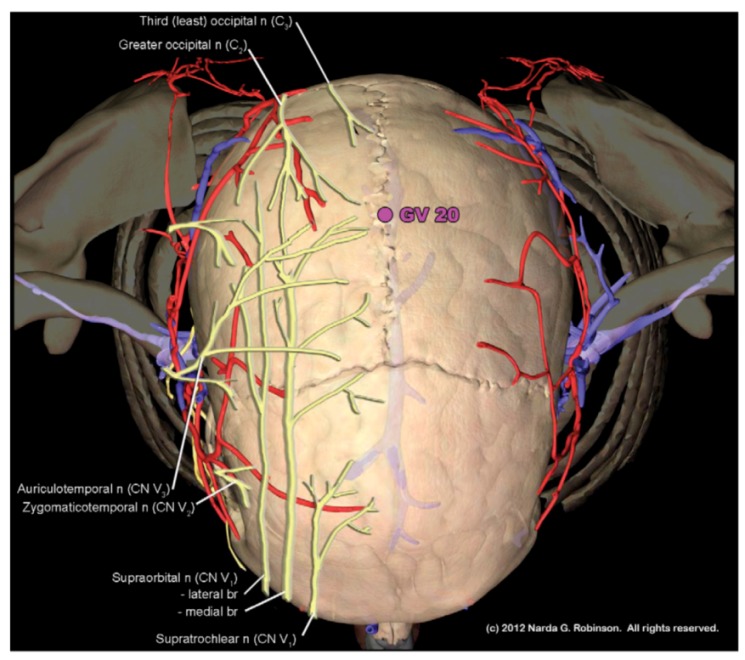
GV 20, known as Bai Hui or “Hundred Convergences” is located at near the vertex of the skull. The term “Hundred Convergences” connotes the meeting place of trigeminal and upper cervical nerves at this site. (Image courtesy of Narda G. Robinson, DO, DVM, MS and Teton NewMedia. From [17]).

**Figure 4:**
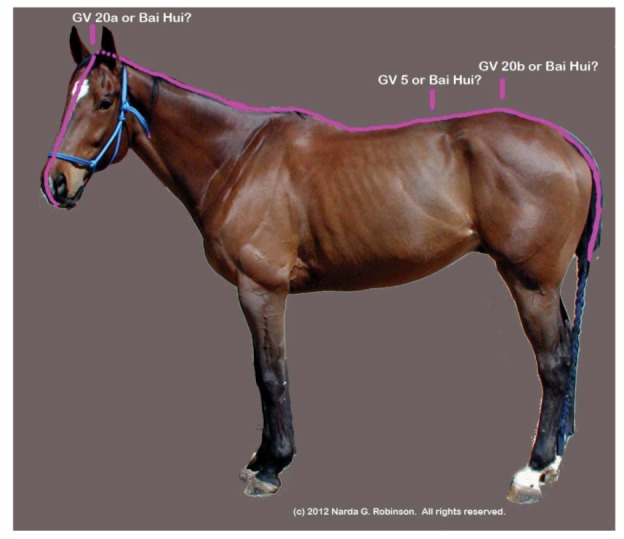
Translational acupuncture research studies citing Bai Hui as one of the points stimulated may be referring to GV 20a, GV 20b, or GV5. The resultant confusion imposes hurdles for researchers who attempt to replicate the study in the same or other species, clinicians who try to achieve the outcomes claimed in the research trial, and physiologists who strive to interpret the mechanisms of action of Bai Hui stimulation. (Image courtesy of Narda G. Robinson, DO, DVM, MS and Colorado State University).

The Bai Hui point illustrates the issue. Bai Hui in humans refers to Governor Vessel 20 (GV 20, Bai Hui or “Hundred Convergences”) near the vertex of the skull (see Figure 3.) On the other hand, TCVM practitioners may variably place GV 20 at the lumbosacral space and refer to the site as either GV 20b or Bai Hui [30,31,32,33]. Studies where researchers included Bai Hui for analgesia may only refer to the name but not provide the anatomical location, specifying either a cranial or lumbosacral site [34]. Making matters worse, some have equated Bai Hui with Governor Vessel 5 (GV 5, Xuan Shu or “Suspended Pivot”), located between the first and second lumbar vertebrae [35]. Obviously, it becomes difficult to “make heads or tails” out of veterinary acupuncture anatomy when a point such as Bai Hui could be placed on the head, nearer to the tail, or somewhere in between. Figure 4 shows the myriad placements of Bai Hui in the nonhuman animal.

Another source of confusion entails the Back Shu points, also known as “Association Points”. This group along the Bladder channel pairs points with organs based on somatovisceral reflex pathways. However, TCVM and transpositional locations differ, sometimes dramatically [36]. As Panzer noted, “Although [TCVM and transpositional] practitioners generally agree that most of the *zangfu *association points can be designated to the urinary bladder channel where it courses along the back, they widely disagree as to the specific placement of the points along this channel [1].” For example, association points for the equine large intestine have been located at the 17th intercostal space in the TCVM system but at the L4 level in certain transpositional atlases, more in keeping with the human location. The TCVM association point for kidney is placed at L5 in comparison to the transpositional L3 location for horses and L2 for humans. TCVM atlases may omit an association point for the heart entirely or instead locate it over the ascending pectoral muscles. Panzer continues, “The numerous differences among modern [TCVM] charts probably arose through the many centuries of Chinese history, as different charts developed in isolated geographical areas. The discrepancies among [TCVM] charts involve different names given for a specific location, varying locations described for a given point name, and descriptions of points that are particular to some charts and absent from others [1].” 

Nerve traffic connecting the Back Shu points and organs courses through spinal cord segments common to both. However, inconsistencies between TCVM and transpositional Back Shu point locations make confident placement difficult. While detailed anatomic investigation should reveal which segments in which species connect to a given organ, much of this work remains to be completed. Alternatively, as Panzer proposed, one might reconcile some of these discrepancies by recognizing Back Shu points as multisegmental regions. This makes neuroanatomic sense since autonomic nerve signals traveling to and from organs to the spinal cord do span several levels [1,37]. However, humans have one point linked to one organ; currently, veterinarians might learn two or more, depending on whether they follow the transpositional, TCVM, or other acupuncture atlases. 

## 8. Additional Veterinary Acupuncture Points in Question

### 8.1. Head

#### 8.1.1. Maxilla

In veterinary patients, head shape varies both between and within species, as in dogs where muzzles range from the dolichocephalic Collie to the brachiocephalic Pug, with the mesaticephalic beagle sporting an average size muzzle. 

How does snout length affect acupuncture points? The main point affected lies over the infraorbital foramen and infraorbital nerve, at Stomach 2 (ST 2, Sibai or “Four Whites”). TCVM authors place ST 2 over the bifurcation of the angular vein at a site otherwise known by the Chinese name “Sanjiang” (Three Rivers) [38], instead of “Sibai” (Four Whites). Needling Sanjiang instead of Sibai will derive different effects, as one penetrates a large vein and the other accesses a notable sensory nerve. Should one attempt to replicate a human or rodent study [39] that examined the effects of needling ST 2, an equine veterinarian selecting the TCVM-style ST 2 would miss the mark by several centimeters. This may confound the outcomes and lead to mistaken interpretations. 

#### 8.1.2. Temporal Fossa

The temporal fossa forms the bony floor upon which the temporalis muscle resides, along with various nerves, vessels, and fascial structures. Needling any of the Gallbladder (GB) points associated with the muscle addresses tension and trigger points that develop as a consequence of masticating and teeth clenching from stress. The architecture of the masticatory system as a whole (including the muscles of mastication, teeth, maxilla, and mandible) exhibits rich anatomical and functional diversity. In keeping with the concept that structure dictates function, as the size and orientation of muscles changes so does the manner in which an animal recruits forces from those muscles to perform chewing motions.

**Figure 5:**
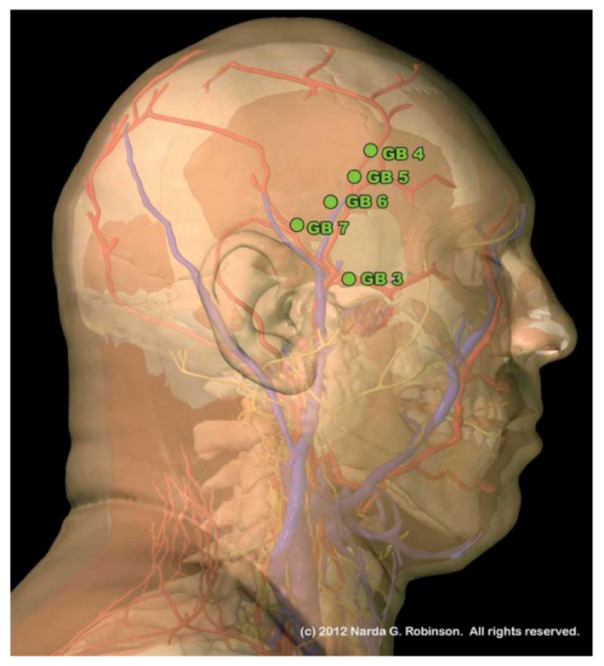
This lateral, layered view of the human illustrates the relationship between the temporalis muscle and a short segment of the Gallbladder channel. Although in humans and ruminants the temporalis muscle is confined to the lateral cranium, in carnivores it may extend up over the head to reach the midline. This would change the configuration of the Gallbladder (GB) channel and the layout of points along it. (Image courtesy of Narda G. Robinson, DO, DVM, MS and Teton NewMedia. From [17]).

The temporalis muscle, a muscle of mastication, varies widely between the dog, ox, horse, and human. In dogs, the temporalis nearly reaches the midline, while in ruminants it remains relegated to a small area on the lateral cranium, pushed aside by the expansive frontal bone that forms the roof of the skull in the ox and pigs. Thus, defining the length and orientation of the Gallbladder channel trajectory in various species must taken into account the surface area of the temporalis muscle atop of which GB 4 through GB 9 reside. Simply drawing a similar line that resembles that of the human misses the meaning of why the points are where they are; *i.e*., the functions and physiologic effects they provide. These GB points in the human most commonly treat temporalis trigger points caused by bruxism (teeth grinding), malocclusion, or other stress and dental disorders. The human image in Figure 5 illustrates the relationship of these points to the temporalis muscle.

#### 8.1.3. Ear

Like the shape of the nose, mandible, and skull, auricular shape differs both between and, at times, within species (e.g., Basset Hound *versus* Cairn Terrier). These anatomical alterations would most directly affect the layout of points in auricular acupuncture, if one agrees that such points exists. Views vary on the mechanisms by which auriculotherapy works, with some authors claiming that Dr. Paul Nogier of France “discovered” a somatotopic map of an inverted fetus on the ear, through which one could influence body function by needling a corresponding site on a fetus pictured on the pinna [40]. More recently, researchers have asserted that auricular acupuncture works through cranial and upper cervical nerve stimulation, as the pinna carries a rich sensory nerve supply. Perhaps most importantly, the auricular branch of the vagus nerve supplies the auricular concha and the external auditory meatus as well as tissue linking the internal and external parts of the ear. Stimulation of regions supplied by this branch influence parasympathetic tone; functionally, this territory overlaps with the organs contained within the imaginary inverted fetus adopted by auriculotherapists [41,42].

Sketching an inverted canine, feline, bovine or equine fetus on the respective species’ ear would seem to require imagination and hope that what one was drawing bore any relationship with reality. However, comparing the distribution of cranial nerves, specifically the auricular branch of the vagus nerve, may offer meaningful insights into the relative clinical value of auriculopuncture for medical problems such as epilepsy and back pain. Both conditions have spawned auriculotherapy research in humans and dogs, though effects of ear acupuncture in dogs have not been shown to be reliable [43,44]. 

### 8.2. Axial Skeleton

#### 8.2.1. Comparative Anatomy of the Spine

Acupuncture points on the back correspond to named vertebral levels. The mammalian vertebral column, consisting of morphologically differentiated groups, *i.e*., cervical (C), thoracic (T), lumbar (L), sacral (S), and caudal (Cd), may differ between species. Although the number of mammalian cervical vertebrae often remains consistent (with 7 vertebrae), birds and reptiles frequently vary in their number of cervical vertebrae. Also, while the human spinal column usually contains 12 thoracic vertebrae and 5 lumbar, the platypus has 17 thoracic and 2 lumbar (with no sacral or caudal features). The vertebral formula for Linne’s two-toed sloth is 7C, 23 T, 3 L, 8 S, and 4 Cd, while that of the pale-throated three-toed sloth, in contrast, is 9 C, 16 T, 3 L, 6 S, and 11 Cd. The human usually lacks a tail entirely, but nerves in the tail present clinically meaningful opportunities for functional recovery of the spinal cord and control over micturition and defecation with acupuncture stimulation. As such, although one would not find such points on a human acupuncture atlas, veterinary acupuncturists need to learn where and why to activate points related to nerves in the tail.

#### 8.2.2. Neck

As indicated above, most but not all non-humans possess 7 cervical vertebrae. Therefore, the bony substrate upon which to locate transpositional points remains consistent. However a clinically relevant translational issue applies to bursae connected to the nuchal ligament in horses, positioned between the bony processes of the vertebrae and the funicular part of the ligament. Aggressively needling the acupuncture point Governor Vessel 16 (GV 16, Fengfu) in horses, overlying the atlanto-occipital junction, might negatively affect the atlantal bursa. Inflammation of the atlantal bursa, called “poll evil”, may also result from infection (brucellosis, *actinomyces bovis*, parasites), poorly fitting tack, or repeated trauma to the occipital region. On the other hand, recognizing tenderness to palpation in this region might inspire treatment with acupuncture, laser, or massage in order to alleviate pain and reduce inflammation. The similar condition of fistulous withers involves supraspinous bursae over the most prominent spinous processes in the cranial thorax. One should thus employ caution when needling GV points over the withers (noted as GV 11, GV 12, GV 13 [45]).

#### 8.2.3. Back

Caudal to the cervical region, the remaining regions of the spine pose numbering challenges for the transposition of points from human to nonhuman due to varying vertebral formulae. This most directly affects the Governor Vessel and Bladder channels. Thus, in addition to incongruities between TCVM and transpositional Back Shu point locations in nonhumans, the fact that many nonhumans simply have more vertebrae means that either the channels have to be “stretched” to fit the same number of points on a longer spine, or more points should be added.

#### 8.2.4. Tail

The tail offers a neuroanatomic entryway for stimulation of neurologic recovery, not available in humans. Tail paresis or neuropraxia frequently accompanies spinal cord injury. Acupressure or needle activation applied to the tip of the tail sends somatic afferent impulses through the most distal spinal cord segments and provides a handy barometer through which to gauge nerve function and recovery in the most caudal spinal cord segments. As movement returns in the tail, fuller and more normal back function often follows. An additional application for this “Tip of Tail” point is fecal incontinence. The client is asked to perform acupressure on the tip of the tail three times daily for five repetitions, and observe the anal sphincter for signs of contraction. In this way, the sphincter gets “toned” and may regain some voluntary function.

#### 8.2.5. Mammae

Mammary gland differences also cause questions for point placement. In the human, Stomach 17 (ST 17, Ru Zhong, “Breast Center”) coincides with the nipple and, as such, needling and moxibustion are proscribed at this location. However, mammae don’t exist in birds, nor does the umbilicus (also forbidden to needle in humans), so do restrictions disappear or change across species? The sow has up to eighteen mammae, while the cow’s mammae occur at the caudal end of the trunk (*i.e.*, the udder), as opposed to the primate’s. Thus, while one might locate ST points according to the distribution of ventral rami from spinal nerves, the functional significance of these points would need to similarly shift. 

### 8.3. Appendicular Skeleton

### 8.3.1. Thoracic and Pelvic Limbs

Many animals differ in body posture and stance (bipedal or quadrupedal). Digits vary as well, reducing down one in the horse. While human primates use their digits to manipulate objects, cats excel at climbing and scratching and dogs at digging. A horse’s feet excel at bearing loads weighing hundreds of pounds (including her own body weight) over rough and rocky terrain.

Pedal peculiarities pose problems for point placement based on neuroanatomical similarity. Absent digits mean that the nerves, vessels, bones, and tendons by which one isolates acupuncture points are missing as well. As such, points such as LI 1, LU 11, TH 1, HT 9, and SI 1 from the human hand, or SP 1, LR 1, ST 45, GB 44, and BL 67 from the foot can find no similar anatomical residence on the one-digit equine foot.

Unfortunately, these anatomical facts have not stopped some authors from imagining the presence of all human points in horses, despite such major differences [46]. However, most end-points of the channels that have typically been placed on the acral equine digit neither structurally nor functionally correspond to those found in the human [47].

Furthermore, missing digits raise nomenclature questions. Without a thumb, and therefore without a structure upon which to justifiably place LU 11 and LU 10, where does the Lung channel begin in the horse? Where does the LI channel end without an index finger? Instead of renumbering the entire channel, one may resolve the issue at least in part by restoring descriptive titles to point names. For example, one could call LU 9 “Great Abyss”, referring to its location over a joint space instead of its sequence along the Tai Yin of the Arm, or Lung channel trajectory. Another option is merely recognizing that it is neither necessary nor anatomically justified to find places for all human points in nonhumans. 

**Figure 6:**
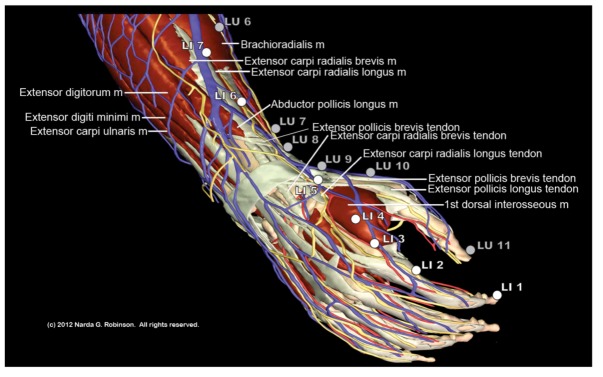
Comparative anatomy calls into question the transposition of many points from the human hand and foot to the equine digit. This image highlights distal points on the Large Intestine (LI) and Lung (LU) channels, all of which should likely be omitted in the horse. Specifically, this entails LI 1 through LI 4, LU 10, and LU 11. (Image courtesy of Narda G. Robinson, DO, DVM, MS and Teton NewMedia. From [17]).

Figure 6 offers an example of the number of points from two digits, the thumb and forefinger, that lack a rational reason to exist in the horse due to comparative anatomy characteristics.

### 8.4. Myofascial Trigger Points

Information about trigger point dysfunction and treatment entered the human medical literature in the mid-twentieth century. Veterinarians have been writing about trigger points for at least two decades [48]. Myofascial trigger points affect nearly everyone at some point during their lives, causing poorly localized, regional, aching pain in the muscles and joints [49]. Simons *et al.*, define a myofascial trigger point as: “a hyperirritable spot in skeletal muscle that is associated with a hypersensitive palpable nodule in a taut band” [49]. Concomitant dysfunctions include weakness, stiffness, restricted range of motion, and sometimes proprioceptive disturbances, sleep disorders, and autonomic dysfunction. 

The importance of addressing trigger points is clear when one considers the physical, psychological, and financial costs to the patient with undiagnosed and untreated myofascial pain. Patients who have experienced severe pain from conditions such as myocardial infarction, fractures, and renal colic have reported that trigger points can be equally painful [49]. Veterinary patients may develop trigger points after trauma, disc injury, surgery, and when over-exercising during rehabilitation. Dogs who run for long distances on hard surfaces or who must exercise beyond their capacity are prone to trigger point development and irritation. Individuals who lack daily moderate exercise and then are exposed to highly strenuous bouts are more prone to develop trigger points than those who exercise on a regular basis [50]. Precipitating factors in horses include improper foot care, poor saddle fit, repetitive strain movements, excessive activity, and sustained confinement. Myofascial trigger points in the equine patient can cause recurring lameness, a sore back, and loss of flexibility [51,52].

Animals with unrecognized myofascial pain from trigger points may undergo costly diagnostics, unnecessary surgery, failed analgesic regimens, and long periods of non-productive inactivity, frustrating clients and veterinarians alike. The suffering endured by the patient, who may be accused of being obstinate, difficult, lazy, or aggressive, is immeasurable. 

Unfortunately, both human and veterinary allopathic medical schools offer insufficient education about the causes and alleviation of myofascial dysfunction. Unawareness of trigger point pathology leads to unnecessary surgery and persistent pain. Fortunately, as scientific research evidence from acupuncture, physical medicine and rehabilitation, and integrative pain medicine permeates other disciplines, more healthcare professionals will learn how to successfully identify and deactivate myofascial trigger points. 

While history suggests that recognition of myofascial trigger points in the Western hemisphere developed relatively recently [53,54], the close correspondence between acupuncture point locations and trigger point locations bespeaks an acknowledgement by ancient acupuncturists of the latter’s existence. According to some, a majority of trigger points coincide with acupuncture points, although this is controversial [55]. 

In humans, active (spontaneously painful) trigger points are commonly found in the postural muscles of the neck, shoulder, and pelvis, as well as in the masticatory muscles. Other frequent sites include the trapezius, scalene, sternocleidomastoid, levator scapulae, and quadratus lumborum [56]. The presence and distribution of trigger points across species relates to not only weight-bearing characteristics but also to the unique activities and environmental pressures to which members of that species are exposed.

Palpating the trigger point may provoke active or latent (previously unappreciated) trigger points and thereby reproduce the pain problem or lameness. Human patients may exclaim, “That’s it! That’s where it is!” Non-human patients often respond with a twitch or move away from the pressure, and occasionally become fractious because of the extreme tenderness elicited. One can treat, or deactivate, trigger points, by applying sustained deep pressure or by inserting a sterile needle into the trigger point, and working the needle back and forth until the muscle tissue softens. 

The fact that “ashi” (also known as “painful”) points vary slightly in location depending on the patient’s particular pain problem reinforces the similarity between dry needling of trigger points and acupuncture treatment of ashi or tender points. Another commonality between trigger points and many acupuncture points pertains to the referred sensations, sometimes painful, that issue from a site when stimulated. In fact, these referral patterns along myofascial “trains” or “chains” linking muscles and fascia in long arcs may have formed the basis for acupuncture channels. For example, deep pressure applied to Gallbladder 21 (GB 21, Shoulder Fountain) in the center of the belly of the anterior border of the trapezius muscle sends referred pain and discomfort in a cranial direction, reaching the mastoid process and arching up and over the ear to the lateral eyebrow. Much of this pain pattern overlaps with the Gallbladder channel’s trajectory [51] (although horses lack the gallbladder organ, they still have been endowed with a Gallbladder channel trajectory. This shows the limitations of the current alphanumeric naming system of channels. The original nomenclature referred to the body surface channels traversed and not an organ. Thus, the original name for the Gallbladder channel of “Shao Yang of the Leg” referred to a channel that traveled along the lateral aspect of the body, running from head to pelvic limb. It is unclear when or why the channels were paired with organs, but the result has led to misunderstandings and misplaced trust in the ability of all or most points along the channel to benefit the organ affiliate. For example, only one (GB 24) of the forty-four GB points clearly relates to the actual gallbladder or, in the case of the horse, perhaps instead hepatobiliary activities. However, most of the myofascial features, nerves, and vessels associated with the GB line in the human appear in nonhumans, including horses. GB points play prominently in equine pain treatments but their names introduce confusion. A better approach may be reverting to the body surface names for the channels instead of organ associations). 

While physicians and physical therapists that dry needle trigger points in humans enjoy the luxury of having reference works that visually display referred pain patterns specific to most skeletal muscles, veterinarians have no such resource. Historically, this harkens back to the development of acupuncture points and channels, where the “dots” representing acupuncture points formed linear channels that corresponded to a “traveling” sensation when a needle entered a site. Thus, even the anatomic outline of the channels houses within it the wisdom of trigger point referred pain patterns as well as neurovascular routes.

Alas, nonhumans cannot tell their acupuncturist where they feel tingling emanate away from a point. This may explain why ancient veterinary channel diagrams portray acupuncture sites as isolated dots instead of collections of points along linear matrices. Because the pain referral pattern is largely a subjective experience, veterinary acupuncturists may never know for sure whether needling a trigger point in a nonhuman produces the same pain pattern as their primate counterparts. However, growing evidence suggests that the benefits of needling overlap across many species. Although a nonhuman cannot say “that’s it” or “ashi” when a needle enters tender tissue, both humans and veterinary patients tend to show a “jump” sign following trigger point palpation or entry by a needle. That is, the body jumps or winces slightly. In addition, needle insertion may generate a local twitch response, confirming entry into the suspected pathology. Electromyographic study of myofascial trigger points in the horse demonstrates similar aberrancies in electrical activity of dysfunctional muscles as found in humans, further attesting to the similarity across species [51]. 

In lieu of trigger point maps that show referred pain patterns for non-humans, veterinary acupuncturists can at least take comfort in knowing where to find the likely culprits. Trigger points can occur in any muscle and usually occur either in the center of the muscle belly or at myotendinous junctions. These locations house dense concentrations of mechanoreceptors, including muscle spindle cells and Golgi tendon organs, respectively. This anatomic determination works in humans, as well. Thus, even without a set of trigger point charts, any medical practitioner with a solid grasp of anatomy can readily predict where these specific sites of myofascial dysfunction may lurk, then palpate and resolve the tension and pain.

## 9. Conclusions

Moving toward a neuroanatomically accurate veterinary acupuncture system requires rethinking current atlases and embarking upon a systematic analysis of the human points [17] in terms of where, if at all, corresponding sites exist in the nonhuman. The next step will involve performing translational research that examines the physiologic outcomes that arise from stimulating similar points in more than one species.

## References

[1] Panzer R.B. (1993). A comparison of the traditional Chinese *versus* transpositional *Zangfu* organ association acupoint locations in the horse. Am. J. Chin. Med..

[2] Klauder J.V. (1958). Interrelations of human and veterinary medicine. N. Engl. J. Med..

[3] King L.J., Anderson L.R., Blackmore C.G., Blackwell M.J., Lautner E.A., Marcus L.C., Meyer T.E., Monath T.P., Nave J.E., Ohle J., Pappaioanou M., Sobota J., Stokes W.S., Davis R.M., Glaser J.H., and Mahr R.K. (2008). Executive summary of the AVMA One Health Initiative Task Force report. J. Am. Vet. Med. Assoc..

[4] Atlas R.M. (2012). One health: Its origins and future. Curr. Top. Microbiol. Immunol..

[5] Leung Z., Middleton D., Morrison K. (2012). One Health and EcoHealth in Ontario: A qualitative study exploring how holistic and integrative approaches are shaping public health practice in Ontario. BMC Publ. Health.

[6] American Veterinary Medical Association (AVMA), One Health Initiative Task Force (2008). One Health: A New Professional Imperative. http://www.avma.org/onehealth/onehealth_final.pdf.

[7] Hayashi A., Matera J.M., Fonseca Pinto A.C. (2007). Evaluation of electroacupuncture treatment for thoracolumbar intervertebral disk disease in dogs. J. Am. Vet. Med. Assoc..

[8] Laim A., Jaggy A., Forterre F., Doherr M.G., Aeschbacher G., Glardon O. (2009). Effects of adjunct electroacupuncture on severity of postoperative pain in dogs undergoing hemilaminectomy because of acute thoracolumbar intervertebral disk disease. J. Am. Vet. Med. Assoc..

[9] Joaquim J.G., Luna S.P., Brondani J.T., Torelli S.R., Rahal S.C., de Paula Freitas F. (2010). Comparison of decompressive surgery, electroacupuncture, and decompressive surgery followed by electroacupuncture for the treatment of dogs with intervertebral disk disease with long-standing severe neurologic deficits. J. Am. Vet. Med. Assoc..

[10] Mayo E. (2012). Acupuncture and wound healing. AJTCVM.

[11] Gerard P.S. (1995). Images in clinical medicine: Acupuncture-like fragments. NEJM.

[12] Imray T.J., Hiramatsu Y. (1975). Radiographic manifestations of Japanese acupuncture. Radiology.

[13] Robinson N.G. Gold bead implants—Medicine or Malpractice?. http://csuvets.colostate.edu/pain/Articlespdf/GoldBeadImplants.pdf.

[14] MacPherson H. (1999). Fatal and adverse events from acupuncture: Allegation, evidence, and the implications. J. Altern. Complement. Med..

[15] Von Riedenauer W.B., Baker M.K., Brewer R.J. (2007). Video-assisted thorascopic removal of migratory acupuncture needle causing pneumothorax. Chest.

[16] Yokogushi K. Embedded needles in acupuncture: Case report and review of the literature. http://www.medicalacupuncture.org/aama_marf/journal/vol15_3/case4.html.

[17] Robinson N.G. Interactive Medical Acupuncture Anatomy.

[18] Wang S.-M., Kain Z.N., White P.F. (2008). Acupuncture analgesia: II. Clinical considerations. Acupunct. Analg..

[19] Takahashi T. (2011). Mechanism of acupuncture on neuromodulation in the gut—A review. Neuromodulation.

[20] White A. (2009). Editorial board of acupuncture in medicine. Western medical acupuncture: A definition. Acupunct. Med..

[21] Lindley S., Cummings T.M. (2006). Chapter 3. Acupuncture—What Is It and How Does It Work?. Essentials of Western Veterinary Acupuncture.

[22] Zhang Z.-J., Wang X.-M., McAlonan G.M. (2011). Neural acupuncture unit: A new concept for interpreting effects and mechanisms of acupuncture. Pflugers Arch..

[23] Zhang Y., Zhang R.X., Zhang M., Shen X.Y., Li A., Xin J., Ren K., Berman B.M., Tan M., Lao L. (2012). Electroacupuncture inhibition of hyperalgesia in an inflammatory pain rat model: Involvement of distinct spinal serotonin and norepinephrine receptors subtypes. Br. J. Anaesth..

[24] Leung A.Y., Kim S.J., Schulteis G., Yaksh T. (2008). The effect of acupuncture duration on analgesia and peripheral sensory thresholds. BMC Complement. Altern. Med..

[25] Chae Y., Lee H., Kim H. (2009). The neural substrates of verum acupuncture compared to non-penetrating placebo needle: An fMRI study. Neurosci. Lett..

[26] Zeng F., Qin W., Ma T., Sun J., Tang Y., Yuan K., Li Y., Liu J., Liu X., Song W. (2012). Influence of acupuncture treatment on cerebral activity in functional dyspepsia patients and its relationship with efficacy. Am. J. Gastroenterol..

[27] Li J., Li J., Chen Z., Liang F., Wu S., Wang H. (2012). The influence of PC6 on cardiovascular disorders: A review of central neural mechanisms. Acupunct. Med..

[28] Beissner F., Deichmann R., Henke C., Bär K.J. (2012). Acupuncture—Deep pain with an autonomic dimension?. Neuroimage.

[29] Schippers R. (1994). Some Aspects of Horse Acupuncture in China in the Middle Ages. Argos.

[30] Skarda R.T., Muir W.W. (2003). Comparison of electroacupuncture and butorphanol on respiratory and cardiovascular effects and rectal pain threshold after controlled rectal distention in mares. Am. J. Vet. Res..

[31] Xie H., Colahan P., Ott E.A. (2005). Evaluation of electroacupuncture treatment of horses with signs of chronic thoracolumbar pain. J. Am. Vet. Med. Assoc..

[32] Jaiswal S., Kumar A. (2005). Clinico-Biochemical effects of electroacupuncture in goats. Indian J. Vet. Surg..

[33] Rungsri P., Trinarong C., Rojanasthien S., Xie H.S., Piransan U. (2009). The effectiveness of electro-acupuncture on pain threshold in sport horses with back pain. AJTCVM.

[34] Lee S.E., Seo J.M., Liu J.Z., Hong M.-S., Lee Y.-W., Lee J.-Y., Song K.-H., Kim D.-H. (2006). The comparison on changes of the body heats in electroacupuncture analgesia and anesthesia by ketamine hydrochloride in dogs. Am. J. Chin. Med..

[35] Kim D.H., Cho S.H., Song K.H., Lee S.-E., Lee S.-H., Kwon G.-O., Kim I.-B., Kim Y.-C., Cho J.-H., Kwon Y.-Y. (2004). Electroacupuncture analgesia for surgery in cattle. Am. J. Chin. Med..

[36] Xie H., Preast V. (2007). Xie’s Veterinary Acupuncture.

[37] Travagli R.A., Hermann G.E., Browning K.N., Rogers R.C. (2006). Brainstem circuits regulating gastric function. Annu. Rev. Physiol..

[38] Xie H., Preast V. (2007). Xie’s Veterinary Acupuncture.

[39] Takayama S., Seki T., Nakazawa T., Aizawa N., Takahashi S., Watanabe M., Izumi M., Kaneko S., Kamiya T., Matsuda A. (2011). Short-term effects of acupuncture on open-angle glaucoma in retrobulbar circulation: additional therapy to standard medication. Evid. Based Complement. Altern. Med..

[40] Frank B.L., Soliman N. (1998). Shen Men: A critical assessment through advanced auricular therapy. Med. Acupunct..

[41] He W., Rong P.-J., Li L., Ben H, Zhu B, Litscher G. (2012). Auricular acupuncture may suppress epileptic seizures via activating the parasympathetic nervous system: A hypothesis based on innovative methods. Evid. Based Complement. Altern. Med..

[42] Lee S.-E., Song K.-H., Liu J., Kwon H.J., Youn S.B., Lee Y.W., Cho S.H., Kim D.H. (2004). The effectiveness of auriculoacupoint treatment for artificially induced acute hepatic injury in dogs. Am. J. Chin. Med..

[43] Still J. (1990). A clinical study of auriculotherapy in canine thoracolumbar disc disease. J. S. Afr. Vet. Assoc..

[44] Panzer R.B., Chrisman C.L. (1994). An auricular acupuncture treatment for idiopathic canine epilepsy: A preliminary report. Am. J. Chin. Med..

[45] Xie H., Preast V. (2007). Xie’s Veterinary Acupuncture.

[46] Thoresen A.S. (2011). Outcome of horses with sarcoids treated with acupuncture at a single Ting point: 18 cases (1995–2009). AJTCVM.

[47] Kim D.H., Liu J.Z., Lee J.Y., MacManus P., Jennings P., Darcy K., Burke F., Rogers P.A.M. (2006). Acupuncture treatment of torticollis in a foal. Korean J. Vet. Res..

[48] Janssens L.A.A. (1991). Trigger points in 48 dogs with myofascial pain syndromes.

[49] Simons D.G., Travell J.G., Simons L.S. (1999). Travell & Simons’ Myofascial Pain and Dysfunction: The Trigger Point Manual, Volume 1 Upper Half of Body.

[50] Meagher J. (1985). Beating Muscle Injuries for Horses.

[51] Macgregor J., von Schweinitz D.G. (2006). Needle electromyographic activity of myofascial trigger points and control sites in equine cleidobrachialis muscle—An observational study. Acupunct. Med..

[52] Rungsri P., Trinarong C., Rojanasthien S., Xie H., Pirunsan U. (2009). The effectiveness of electro-acupuncture on pain threshold in sport horses with back pain. Am. J. Trad. Chin. Vet. Med..

[53] Frank E.M. Myofascial Trigger Points and Acupuncture. Proceedings of the 32nd World Small Animal Veterinary Association Congress.

[54] Melzack R., Stillwell D.M., Fox E.J. (1977). Trigger points and acupuncture points for pain: Correlations and implications. Pain.

[55] Janssens L.A.A., Schoen A.M. (2001). Trigger Point Therapy. Veterinary Acupuncture: Ancient Art to Modern Medicine.

[56] Mackley R.J. (1990). The role of trigger points in the management of head, neck, and face pain. Funct. Orthod..

